# Psychotropic medication use in pediatric population during COVID‐19 pandemic

**DOI:** 10.1111/acps.13483

**Published:** 2022-08-03

**Authors:** Ilari Kuitunen

**Affiliations:** ^1^ Department of Pediatrics, Institute of Clinical Medicine University of Eastern Finland Kuopio Finland; ^2^ Department of Pediatrics Mikkeli Central Hospital Mikkeli Finland

## PEER REVIEW

The peer review history for this article is available at https://publons.com/publon/10.1111/acps.13483.

Strict lockdown strategies, including school and day care closures, were used to prevent the spreading of COVID‐19 in the spring 2020. As the pandemic continued, the role of the children was not considered as the main driver of the pandemic, and more discussion on the collateral damage of the restrictions against children emerged. This led to reopening of schools in May 2020 in Finland. Since then, children aged less than 13 have attended school without restrictions or masks in Finland.

Studies have reported increased anxiety and depression disorders during the lockdowns and restriction periods.^1^, ^2^ The use of psychiatric services has increased in children and adolescents.^2^, ^3^ Previous reports from adolescents and adults have presented increased psychotropic medication consumption during the pandemic.^4^ Therefore, the aim of this paper is to examine the psychotropic medication consumption in Finnish children (age 6–12 years, attending to lower elementary school) during COVID‐19 pandemic.

All psychotropic medication purchases were retrieved for children aged 6–12 years from the Pharmacy Register, which is maintained by the Finnish Social Insurance Institute, the register does not hold individual patient level data openly available. The first (April 2020 to March 2021) and second (April 2021 to March 2022) pandemic years were compared with reference year (April 2019 to March 2020). Psychotropic medications were included based on the anatomic therapeutic chemical classification and psycholeptics, antidepressants, and psychostimulants were included. Three most used in each class are presented separately. Finland has publicly funded healthcare and children social insurance, which covers part of the medication purchases from pharmacies.

Prevalence of psychotropic medication users in yearly quarters and annual incidence (incidence calculated as number of medication purchases divided by the population size and multiplied by 1000) of medication purchases per 1000 children was calculated. Medication user is classified as psychotropic medication purchase during the selected period. Incidence rate ratios with 95% confidence intervals were used for comparisons. Comparisons are made between years and yearly quarters (Q1, Q2, Q3, and Q4). This study used open‐access data. Due retrospective and register‐based cross‐sectional design, research permission was not needed.

**FIGURE 1:**
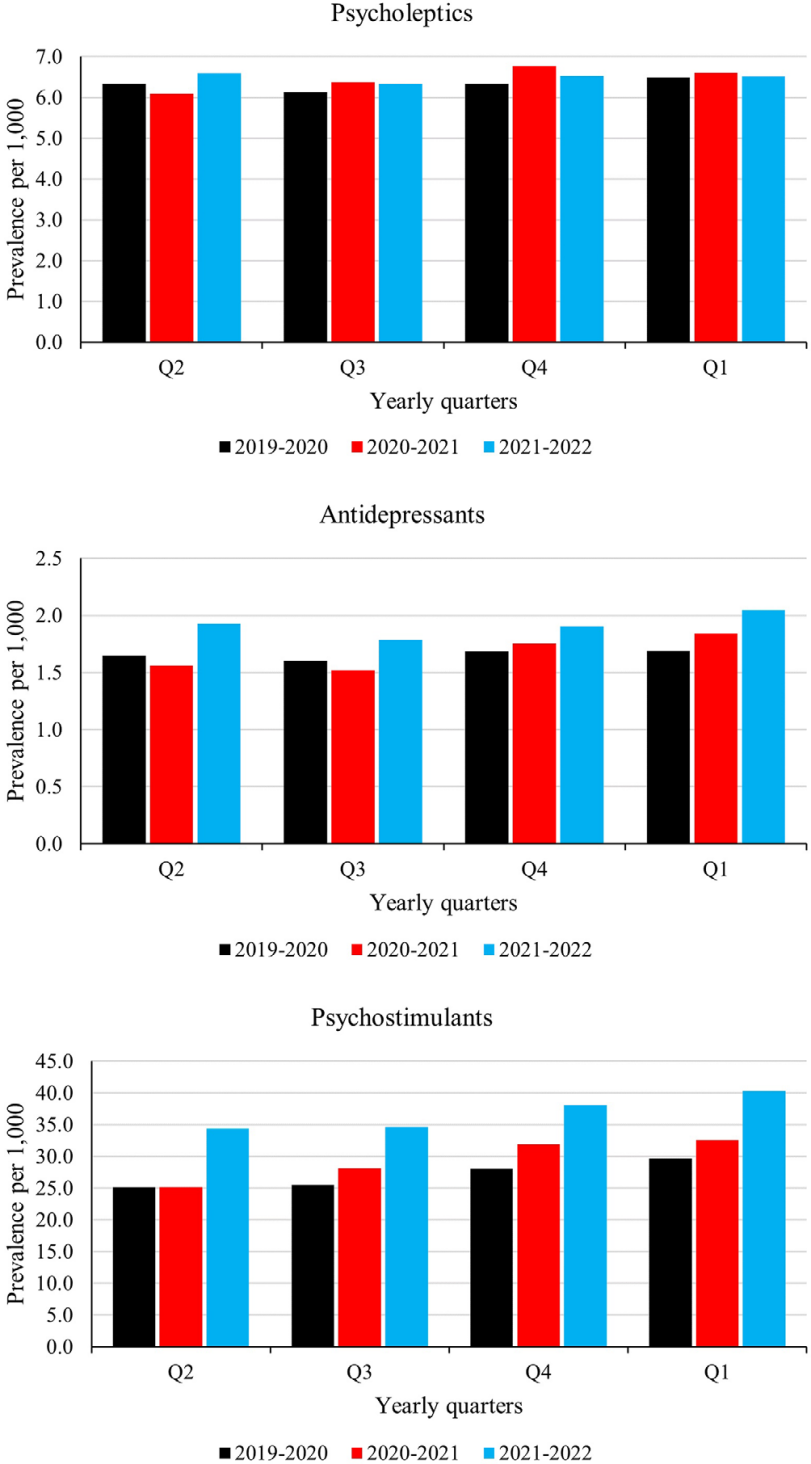
Psychotropic medication user prevalence per 1000 children aged 6–12 years in Finland from 2019 to 2022. Yearly quarters of the first pandemic year (April 2020 to March 2021) and the second pandemic year (April 2021 to March 2022) compared with pre‐pandemic year April 2019 to March 2020.

A total of 402,091 psychotropic medication purchases were included. The prevalence of psychotropic medication users was lower in Q2 of 2020 (strict lockdown period) than in the reference year (Figure 1). Since then, the prevalence has been above the reference years in antidepressants and psychostimulants, whereas psycholeptics prevalence has remained stable.

The most purchased medication was methylphenidate (Table S1). Highest increase was in lisdexamfetamine purchases during the second pandemic year (+54%). Antidepressant use increased by 14% in the second pandemic year. The overall incidence of psycholeptic purchases remained unchanged during the study period.

Continuation of the COVID‐19 pandemic has led to increase in the prevalence of antidepressant and psychostimulant medication users. There was no overall increase in the incidence of psycholeptic medication purchases as these are used in children for severe mental health or aggression issues. However, our results are in line with previous reports, that depression and anxiety has increased in children during the pandemic.^1^, ^2^ This was seen as an increase in the use of antidepressant during the second pandemic year. The increase was smaller than previously reported in adolescents and adults.^4^

The prevalence of psychostimulant medication users in Finnish has continued to increase during the pandemic and at the end of our study period 4% of the children used psychostimulants. This was unsurprising as previous reports have described the continued increase in ADHD diagnoses and psychostimulant use in children and adolescents.^5^

First limitation is the lack of the doses in medication purchases, which meant that defined daily dose calculations were not made and incident medication use could not be separated from continuous medication usage. This would have required individual patient level data, which would be available in late 2023 earliest. Second is the lack of information on primary/secondary/tertiary, and private care visit rates, as these data are not available until 2023. The visit information would have allowed to analyze whether the prescription trends were affected by limited access to healthcare. Although, it must be noted that the lack of visit rates does not affect the prescription statistics as the prescriptions are reported to the pharmacy register from pharmacies regardless of where the prescription was ordered. Third limitation is the rather wide age grouping (from 6 to 12 years), as this is defined by the register holder and could not be changed. The broad age range may have underestimated the psychotropic medication user prevalence changes in the older children (10–12 years) as, those have been affected more mentally during the pandemic, and medication user prevalence increases typically with age. The main strength is the nationwide register covering all Finnish pharmacies and the universal social insurance which means that our results are nationwide.

In conclusion, the use of psychotropic medication increased during the pandemic and especially in the second pandemic year. This increase was seen in the purchases of antidepressants and psychostimulants. These results can be used in designing policies to improve youth health in crisis time and how we overcome the increased mental health issues.

## Supporting information


**TABLE S1**: Yearly incidence of medication purchases per 1000 children. The first pandemic year (April 2020 to March 2021) and the second pandemic year (April 2021 to March 2022), compared with pre‐pandemic reference year (April 2019 to March 2020). Comparisons made by incidence rate ratios (IRR) with 95% confidence intervals (CI).

## Data Availability

The data that support the findings of this study are available from the corresponding author upon reasonable request.
