# Supplementary material for: Psychotropic medication use in pediatric population during COVID‐19 pandemic
Source: Acta Psychiatr Scand. 2022 Aug 3;146(4):381–3. doi: 10.1111/acps.13483 (PMC9353282; doi:10.1111/acps.13483)
Supplement: Supplementary file 1 — TABLE S1: Yearly incidence of medication purchases per 1000 children. The first pandemic year (April 2020 to March 2021) and the second pandemic year (April 2021 to March 2022), compared with pre‐pandemic reference year (April 2019 to March 2020). Comparisons made by incidence rate ratios (IRR) with 95% confidence intervals (CI). [file ACPS-146-381-s001.docx]

**Table S1** Yearly incidence of medication purchases per 1,000 children. The first pandemic year (April 2020 to March 2021) and the second pandemic year (April 2021 to March 2022), compared to pre-pandemic reference year (April 2019 to March 2020). Comparisons made by incidence rate ratios (IRR) with 95% confidence intervals (CI)

|  | Pre-pandemic year | | First pandemic year | | | | Second pandemic year | | | |
| --- | --- | --- | --- | --- | --- | --- | --- | --- | --- | --- |
| Medication | n | inc | n | inc | IRR (CI) | n | | inc | IRR (CI) |  |
| N05* Pscyholeptics | 17,952 | 41.5 | 17,751 | 41.0 | 0.99 (0.97-1.01) | 18,115 | | 41.8 | 1.01 (0.99-1.03) |  |
| N05AX08 Risperidone | 11,193 | 25.8 | 10,762 | 24.9 | 0.96 (0.94-0.99) | 11,163 | | 25.8 | 1.00 (0.97-1.02) |  |
| N05AX12 Aripiprazole | 3,038 | 7.0 | 3,244 | 7.5 | 1.07 (1.02-1.12) | 3,471 | | 8.0 | 1.14 (1.09-1.20) |  |
| N05AH04 Quetiapine | 1,057 | 2.4 | 1,058 | 2.4 | 1.00 (0.92-1.09) | 984 | | 2.3 | 0.93 (0.85-1.02) |  |
| N06A* Antidepressants | 4,260 | 9.8 | 4,234 | 9.8 | 0.99 (0.95-1.04) | 4,838 | | 11.2 | 1.14 (1.09-1.18) |  |
| N06AB03 Fluoxetine | 2,078 | 4.8 | 1,964 | 4.5 | 0.95 (0.89-1.01) | 2,393 | | 5.5 | 1.15 (1.09-1.22) |  |
| N06AB06 Sertraline | 1,728 | 4.0 | 1,815 | 4.2 | 1,05 (0.98-1.12) | 2,023 | | 4.7 | 1.17 (1.10-1.25) |  |
| N06AB10 Escitalopram | 259 | 0.6 | 295 | 0.7 | 1.14 (0.96-1.35) | 386 | | 0.7 | 1.10 (0.93-1.31) |  |
| N06B* Psychostimulants | 98,593 | 227.7 | 106,939 | 247.0 | 1.08 (1.08-1.09) | 129,409 | | 298.9 | 1.31 (1.30-1.32) |  |
| N06BA04 Methylphenidate | 86,273 | 199.2 | 93,303 | 215.5 | 1.08 (1.07-1.09) | 112,939 | | 260.8 | 1.31 (1.30-1.32) |  |
| N06BA12 Lisdexamfetamine | 6,833 | 15.8 | 8,323 | 19.3 | 1.22 (1.18-1.26) | 10,551 | | 24.4 | 1.54 (1.50-1.59) |  |
| N06BA09 Atomoxetine | 5,307 | 12.3 | 5,030 | 11.6 | 0.95 (0.91-0.99) | 5,573 | | 12.9 | 1.05 (1.01-1.09) |  |

inc=incidence
